# Editorial: Pharmacology Education Special Issue

**DOI:** 10.1002/prp2.70263

**Published:** 2026-05-08

**Authors:** Jennifer A. Koenig, Steven J. Tucker

**Affiliations:** ^1^ School of Medicine University of Nottingham Nottingham UK; ^2^ University of Aberdeen Aberdeen UK

**Keywords:** curriculum, inclusive education, pharmacology education

Research and scholarship in pharmacology education is in good shape. Since 2021 when PR&P announced the establishment of Pharmacological Education as a new topical section of the journal [1], the professional societies have put on many very popular education symposia at their annual meetings, have strong educator networks, and the IUPHAR‐Education section has expanded to include online meetings, newsletters, and social media communications. Together this support for the research and scholarship of pharmacology education has promoted significant improvement in pharmacology education and enhanced the careers of pharmacology educators.

The impetus for this Pharmacology Education special issue started with a symposium on inclusive pharmacology education at Pharmacology 2022, the BPS annual meeting, and publication of the Principles for inclusive implementation of the undergraduate pharmacology core curriculum [2]. A call for papers took place in 2024–2025 resulting in seven papers which cover the breadth of inclusive pharmacology education.

But what is inclusive pharmacology education? It is an approach to education that considers the diversity of learners, their interests and backgrounds, recognizes the need for accessibility and should be implicit in all aspects of pedagogical practice from curricular design through to assessment. In short, it is an education which meets the needs of all learners [3]. There are many aspects to designing and delivering an inclusive curriculum and there are guides and frameworks to help think through how this philosophy of education is borne out in practice, such frameworks include Universal Design for Learning and various Inclusive Curriculum Frameworks [4, 5].

One aspect of inclusive curriculum is to recognize that students need to be able to solve problems through a sound understanding of the underpinning concepts and there has been a move away, over the past 30 years, from a curriculum stuffed full of facts to one that is based on big ideas otherwise known as core concepts. The paper by Michael and White [6] places the development of core concepts in physiology and pharmacology into perspective and makes useful, practical suggestions for structuring a pharmacology education around core concepts or big ideas [6]. The IUPHAR Core Concepts of Pharmacology project is making great progress in defining, unpacking, and understanding how students understand these concepts [7, 8, 9].

A curriculum is defined as not only the list of learning outcomes or the topics covered, but it also encompasses the hidden curriculum, that is, the implicit knowledge, understanding and ways of working. The hidden curriculum is often propagated unseen and unchallenged until a groundswell of opinion in wider society changes social perspectives. The pandemic and Black Lives Matter protests of 2020 led to a re‐evaluation of many clinical algorithms such as spirometry measurements [10] and kidney function measurements using eGFR [11]. In pharmacology, much of the research around ethnic differences focusses on genetic polymorphisms [12] and this is reflected in teaching that emphasizes differences in variants of drug metabolizing enzymes and transporters between ethnic groups. However, genetics education research has shown over several years, that there are several misconceptions about genetic variation and ethnicity and this work has highlighted the importance of being very clear in teaching, that certain SNPs are not exclusive to one ethnic group and natural variation of humans around the world does not underpin social constructions of race or ethnicity. Koenig et al. [13] describe design of a tutorial about inter‐individual variation in pharmacokinetics that challenges misconceptions about ethnicity and population descriptors. By demystifying this myth around polymorphisms and ethnicity, the next generation of pharmacologists are equipped and empowered to carry out research and teaching in an inclusive manner.

Another aspect to inclusive education is to use a mixture of pedagogical approaches and new technologies can open up possibilities catering to broader range of learners. Trying out these new approaches and technologies and reporting on their advantages and limitations is an important feature of pharmacology education research and scholarship. Virtual and augmented reality technologies are increasingly attractive and an article by Khaiyam et al. [14] describes the use of virtual reality—the meta‐horizon workroom—in a problem‐based learning setting.

Clear and consistent language is important, and assessment can prove challenging when educators and students are not clear about how important terms are used. In their paper on the language of assessment, Kelly‐Laubscher et al. [15] found that examiners did not always agree on how common action verbs such as describe, discuss, and explain should be interpreted. Reaching agreement on this amongst examiners is vital, as is the next step of ensuring that students are also clear about what these words are telling them to write about in an assessment. Another important point in this paper is that there should be an alignment between the learning outcomes and the assessment through the use of commonly understood language.

Understanding what students know and how they feel about their education is an important first step in ensuring the course and, in particular, the learning demand are appropriate. Student feedback and staff‐student dialogue can be very powerful in making changes that enhance inclusivity in learning. In the context of a medical pharmacology course, Smith et al. [16] found that it was better to introduce a smaller number of drugs and focus on those that are most relevant than to try to cover too many different drugs with the resulting sense of overwhelm. They also noted that integrating the pharmacology and placing it within clinical contexts was a positive solution and provided dimensional context to the learning material.

It is important to recognize that students don't only learn pharmacology in a pharmacology course; they also develop skills which are transferable to future careers. Designing a curriculum that intentionally develops these skills is an important aspect of an inclusive curriculum. Yeh and Richardson [17] describe an iterative approach to building a curriculum around skills development in graduate education. As they note, it can be difficult to design a course that meets the needs of all students as their ambitions and motivations can vary so much. Striking a balance between student choice and encouraging students to find out and appreciate those unknown unknowns can be challenging but is an effective way to develop autonomous learning practices amongst students.

In summary, this Pharmacology Education special issue presents an array of perspectives in taking an inclusive approach to teaching and learning and curriculum design and development. It also provides a series of actionable and adaptable approaches that can be integrated into educator practices across and beyond the spectrum of pharmacology teaching, learning, and assessment, equipping educators with examples that will develop future scientists with a more accurate, sensitive, and realistic world view. Taking this forward, there is great potential to use the existing national and international networks to exploit the potential for cross‐institution collaboration, which would strengthen our understanding of how pedagogical approaches play out in practice in a range of environments.

## Author Contributions

**Jennifer A. Koenig:** conceptualization, writing – original draft, writing – review and editing. **Steven J. Tucker:** conceptualization, writing – original draft, writing – review and editing.

## Data Availability

The authors have nothing to report.
